# Technological Approaches in the Analysis of Extracellular Vesicle Nucleotide Sequences

**DOI:** 10.3389/fbioe.2021.787551

**Published:** 2021-12-23

**Authors:** Tine Tesovnik, Barbara Jenko Bizjan, Robert Šket, Maruša Debeljak, Tadej Battelino, Jernej Kovač

**Affiliations:** ^1^ Institute for Special Laboratory Diagnostics, University Medical Centre Ljubljana, University Children’s Hospital, Ljubljana, Slovenia; ^2^ Department of Pediatric Endocrinology, Diabetes and Metabolic Diseases, University Medical Centre Ljubljana, University Children’s Hospital, Ljubljana, Slovenia; ^3^ Faculty of Medicine, Chair of Paediatrics, University of Ljubljana, Ljubljana, Slovenia

**Keywords:** extracellular vesicles (EVs), DNA, RNA, biomarkers, nucleotide sequences detection, therapeutics

## Abstract

Together with metabolites, proteins, and lipid components, the EV cargo consists of DNA and RNA nucleotide sequence species, which are part of the intracellular communication network regulating specific cellular processes and provoking distinct target cell responses. The extracellular vesicle (EV) nucleotide sequence cargo molecules are often investigated in association with a particular pathology and may provide an insight into the physiological and pathological processes in hard-to-access organs and tissues. The diversity and biological function of EV nucleotide sequences are distinct regarding EV subgroups and differ in tissue- and cell-released EVs. EV DNA is present mainly in apoptotic bodies, while there are different species of EV RNAs in all subgroups of EVs. A limited sample volume of unique human liquid biopsy provides a small amount of EVs with limited isolated DNA and RNA, which can be a challenging factor for EV nucleotide sequence analysis, while the additional difficulty is technical variability of molecular nucleotide detection. Every EV study is challenged with its first step of the EV isolation procedure, which determines the EV’s purity, yield, and diameter range and has an impact on the EV’s downstream analysis with a significant impact on the final result. The gold standard EV isolation procedure with ultracentrifugation provides a low output and not highly pure isolated EVs, while modern techniques increase EV’s yield and purity. Different EV DNA and RNA detection techniques include the PCR procedure for nucleotide sequence replication of the molecules of interest, which can undergo a small-input EV DNA or RNA material. The nucleotide sequence detection approaches with their advantages and disadvantages should be considered to appropriately address the study problem and to extract specific EV nucleotide sequence information with the detection using qPCR or next-generation sequencing. Advanced next-generation sequencing techniques allow the detection of total EV genomic or transcriptomic data even at the single-molecule resolution and thus, offering a sensitive and accurate EV DNA or RNA biomarker detection. Additionally, with the processes where the EV genomic or transcriptomic data profiles are compared to identify characteristic EV differences in specific conditions, novel biomarkers could be discovered. Therefore, a suitable differential expression analysis is crucial to define the EV DNA or RNA differences between conditions under investigation. Further bioinformatics analysis can predict molecular cell targets and identify targeted and affected cellular pathways. The prediction target tools with functional studies are essential to help specify the role of the investigated EV-targeted nucleotide sequences in health and disease and support further development of EV-related therapeutics. This review will discuss the biological diversity of human liquid biopsy–obtained EV nucleotide sequences DNA and RNA species reported as potential biomarkers in health and disease and methodological principles of their detection, from human liquid biopsy EV isolation, EV nucleotide sequence extraction, techniques for their detection, and their cell target prediction.

## Introduction

From the perspective of abiogenesis, the beginning of early forms of life started with the synthesis of organic molecules, encapsulation of nucleotide sequence-like molecules, and later RNA molecules inside the lipid-like vesicles (Copley et al., 2007; Chen and Walde, 2010). These formed units with an individual nanocatalytic RNA activity inside the lipid layer vesicles provided the environmental protection of catalytic and genetic material (Lazcano et al., 1988). The early forms of life had to receive the substrates from the environment and remove the toxic products from their interior through a semipermeable membrane or early cell-like transport *via* bursting and fusion of vesicles under turbulent environmental forces. With the development of deoxyribonucleotides and DNA molecules, more stable genomic information was generated and gene units for independent generation biological molecules of self-sufficient units were formed (Kurihara et al., 2011). These protocells were able to replicate and a need for more complex cell-directed intracellular communication has arisen, where vesicles very likely played the important role in intracellular communication. Vesicles were probably one of the first forms of intracellular communication involved in cell adaptation to environmental challenges and allowed the integration of cells in multicellular organisms. This assumption is supported with the interkingdom vesicle communication and its genetic code horizontal transfer, which gave cells an additional developmental advantage to form better environmental adaptation and led to the development of higher forms of life (Kawamura et al., 2017; Correa et al., 2020). The vesicle communication mechanism is evolutionarily preserved and still present from prokaryotes and protozoa to mammals, indicating a highly conserved process of vesicular intracellular communication in all forms of life.

The extracellular vesicles (EVs) in humans and other mammals include exosomes, microvesicles, and apoptotic bodies. All classes of EVs and their heterogeneous subsets differentiate in their biogenesis, diameter, and cargo, but all have a common feature of carrying and transporting parental cell molecules to target cells. Together with metabolites, proteins, and lipid components, the EV cargo consists of DNA and RNA molecules involved in the intracellular communication network regulating specific cellular processes and inducing distinct target cell responses (van Niel et al., 2018; Doyle and Wang, 2019). Human EV nucleotide sequences are one of the most diverse components of EVs and are often investigated as biomarkers and proxies in intracellular communication in association with the pathology of diseases (Koniusz et al., 2016). As biomarkers, EVs provide an insight into the physiological and pathological processes in hard-to-access organs and tissues, where conventional diagnostics requires invasive and risky procedures (Zhou et al., 2020). In the human body, EV intracellular communication ensures normal cell and tissue operation and can be involved in the pathological processes’ suppression. However, EVs can be also involved in pathogenesis promotion (Hanayama, 2021). The pathology of diseases is generally caused by cellular disequilibrium, causing cell stress, and senescence. While the EV biogenesis is altered in pathogenesis, EVs can also spread cell stress signals and promote cell senescence. In addition, EVs are factors involved in developing neurodegenerative diseases (Izadpanah et al., 2018), autoimmunity (Wu et al., 2020), and cancer (Yu et al., 2021). The potential of the liquid biopsy–obtained samples, which could be collected in a non-invasive manner or by low-invasive procedures with the collection of urine, blood, saliva, cerebrospinal fluid, and other body fluids, allows EV isolation for their characterization and detailed analysis (Doyle and Wang, 2019). With the rise of interest in the EV research, the field of EV nucleotide sequence detection and the development of novel methodological technologies have become one of the most exciting fields of the EV research. A variety of novel methodological approaches of EV DNA and RNA sequence detection allows defining the EV biomarkers and recognizing specific EV nucleotide sequences as drivers and regulators of targeted cell processes (Lane et al., 2018; Xu et al., 2020). A huge diagnostic potential of cell-free EV nucleotide sequences results in increased interest in developing clinical diagnostic liquid biopsy platforms for early disease detection and prevention of complications of diseases. Diagnostic companies have already focused on cancer screening and cancer diagnostic applications, but the EV nucleotide sequences have the potential to develop prognostic and diagnostics tests even for other pathological states (Zhou et al., 2020). With the standardization of research approaches and the EV non-invasive early diagnostic test potential, EVs will become standard diagnostic tools (Zhou et al., 2020). EVs with synthetic DNA and RNA and EV-targeted delivery present great potential in gene-directed therapy, targeting specific cells in an organism and inducing the specific phenotypic response. The knowledge of the modified gene expression in cells and tissues with the support of prediction interaction tools could provide the path for developing therapeutic EVs with nucleotide sequences to treat various diseases, even orphan diseases with genetic backgrounds.

In the next chapters, we will briefly sum up the characteristics of EV nucleotide sequence species with their role and basic mechanisms in human cells and organisms. In the continuation, we describe the methods and procedures of EV nucleotide sequence biomarker discovery with methodological advantages and disadvantages and describe the prediction of EV nucleotide sequence cellular functions and their potential as therapeutics.

### EV Nucleotide Sequences

The EV cargo consists of nuclear and mitochondrial DNA, while the EV transcriptome is more diverse and includes different coding and non-coding RNA species (Malkin and Bratman, 2020; Veziroglu and Mias, 2020). The EV DNA and RNA cargo reflect heterogenic EV genomic and transcriptomic profiles of parental cells, and this feature can be used to characterize the EVs as biomarkers and future clinical diagnostic tools (Lázaro-Ibáñez et al., 2019). How the nucleotide sequences are packed into EVs is not well understood, but published studies report different mechanisms involved in nucleotide sequence EV sorting. While the EV cargo release is an energy-consuming process, the cells generally control EV release and cargo loading of specifically selected nucleotide sequence transfer to targeted cells (Malkin and Bratman, 2020; O’Brien et al., 2020). However, the EV DNA and RNA packing can also be non-directed and result in a similar transcriptomic and genomic profile as the parental cell.

### EV DNA

EV DNA is mainly studied in apoptotic bodies, where during the apoptosis process, degraded cell components, including degraded genomic DNA, are packed in apoptotic bodies in the form of short DNA fragments of a few hundred nucleotides (Malkin and Bratman, 2020). The apoptotic body clearance is determined by the apoptotic body diameter, where smaller apoptotic bodies have a longer life span than larger ones, and can sustain in circulating body fluids longer and can transfer EV DNAs to targeted cells. Most apoptotic body EVs and their DNAs are cleared by phagocytosis by cells of the immune system or neighboring cells quickly after cell apoptosis (Malkin and Bratman, 2020). The intensive release of apoptotic bodies is present in late-stage tumors, where hypoxic environments promote the release of more extensive apoptosis- and necrosis-associated onko-EVs with larger DNA fragments (Vagner et al., 2018). These onko-EV degradomes often harbor somatic pro-oncogenic genomic aberrations such as single nucleotide mutations and copy number variations in *TP53*, *KRAS*, and *PTEN* genes frequently altered in cancer and metastasis (Bergsmedh et al., 2001). The transferred onko-EV DNAs can promote horizontal onko-gene transfer, associated with spreading tumor mutations, promoting a pro-oncogenic and pro-metastatic environment in other tissues and harboring novel mutations (Bergsmedh et al., 2001; Kawamura et al., 2017; Amintas et al., 2021).

The reporting of genomic DNA fragments in EVs within the diameter of exosomes and microvesicle EVs is inconsistent. Several studies report the presence of DNA in all subgroups of EVs; some of them report that DNA might not be associated with the parental cell release, but as DNA bonded outside on the surface of EVs in the extracellular space, body fluids, or during sample processing (Lázaro-Ibáñez et al., 2019; Elzanowska et al., 2021; Hur and Lee, 2021). Even if DNA is bonded on the surface of EVs, it can be biologically active and can have its biological function. The EV-transferred DNA can regulate the immune system *via* TLR9 receptors, which detect non-methylated CpG-containing DNA delivered by the endocytotic pathway as part of the anti-pathogen response mechanism (Hemmi et al., 2000) or potentially even modified human DNA (Ye et al., 2017). While the normal tissue cells include germline DNA, with attained somatic variants, the cells and EVs also carry different DNA epigenomic information, which is tissue-specific and contains methylated, oxidated, and other nucleotide modifications produced by cell enzymes and reactive chemical species (Mannavola et al., 2019). Cell and EV DNA epigenome with modification differences reflect environmental and tissue physiological specificity that needs to be investigated.

### EV RNA

Cell transcriptome includes diverse coding and non-coding RNA molecules, which are specific for cell types and tissues. The transcripts are primarily generated with the DNA transcription in the nucleus, where primarily transcripts are processed and trimmed. In the following steps, the transcripts are transported to the cytoplasm, where the RNA molecules are post-transcriptionally processed and modified to the active form of coding or non-coding RNA, which can perform their functions in a cell or can be directed into EVs for EV cell release (Tosar et al., 2021). The heterogeneous cell- and tissue-dependent RNA profile is reflected in EV transcriptome heterogenicity (O’Brien et al., 2020). All RNA species were reported in the EV cargo. However, due to the thermodynamically favorable processes, only small RNA molecules are preferentially abundant in the EV cargo. RNAs are packed into EVs by selective and non-selective EV nucleotide sequence-loading mechanisms (O’Brien et al., 2020; Veziroglu and Mias, 2020). While some studies report “naked” RNA EV loading, the other results suggest RNA EV-directed loading with RNA-binding proteins, even as active regulatory complexes (O’Brien et al., 2020; Veziroglu and Mias, 2020).

Although the EV transcriptomic profiles are concentrated in short RNA sequences, EVs intact mRNA and long ncRNAs (lncRNA) were reported. Even though the mRNAs are not highly represented in EVs due to their size, EV mRNAs were reported in several studies which suggest the intracellular horizontal cell-coding transcriptome transfer (Cha et al., 2020; Bracht et al., 2021). EV lncRNAs, longer than 200 bps with no apparent coding functions, were investigated as biomarkers and mediators involved in oncogenesis as well as tissue repair and regeneration (Kogure et al., 2013; Born et al., 2020). Only a few studies report the EV presence of the larger rRNA units (18S and 28S), while it is inconclusive if these molecules are present due to non-EV RNA contaminations or parts of RNA degradome produced in cell necrosis or apoptosis. Nevertheless, smaller rRNA units, 5S and 5.8S rRNA with a length up to 150 nucleotides (Tosar et al., 2020), whose function is an enhancement of the protein synthesis and stabilization of a ribosomal structure, are commonly reported within EVs (Szymanski et al., 2002). Most of the EV transcriptomic profile belongs to small non-coding RNAs (sncRNAs), where the most abundant sncRNAs are miRNA, yRNA, siRNA, piRNA, and tRNA represented as mature sncRNAs, their precursors, or isoforms. The abundance of sncRNA types varies and is highly related to the sample type, sample processing, and the EV isolation methodology.

The EV research is mainly directed into EV miRNA (microRNA) due to the well-characterized miRNA regulatory function in the cellular transcription and regulation of the mRNA expression to coding proteins. However, miRNA function is even more diverse. miRNAs are 16–24 nucleotides long sncRNA, coded by the miRNA genes which are in the human genome often located in miRNA gene clusters. 2,654 miRNA human sequences and 1,917 miRNA precursors are identified so far and their sequences are listed in miRBase (http://www.mirbase.org/; Release 22.1) (Kozomara et al., 2019). It is estimated that miRNAs can regulate the translation of more than 60% of human mRNAs to proteins (Friedman et al., 2009). The miRNA synthesis starts in the nucleus by polymerase II transcription, where pre-miRNA transcripts are formed. In the nucleus, pre-miRNAs are cleaved with the Drosha-DGCR8 complex and a stem-loop structure, which is transported in the cytoplasm by the exportin complex, is formed. In the cytosol, pre-miRNA is diced by the Dicer protein complex to 3′ and 5′ miRNA strands, and other non-template nucleotide additions (NTA) can be added on the 3′ and 5′ ends of miRNA. One of the formed miRNA strands (5p or 3p) is degraded, while the other miRNA strand is incorporated into Ago proteins and forms the RISC complex (O’Brien et al., 2018). The RISC-miRNA complex can bind on mRNA, mostly on 3′ mRNA and can be involved in the translation regulation with protein translation silencing or mRNA degradation. Given the knowledge of miRNA and prevalence of EV miRNA studies, EV miRNAs have been investigated in most diseases and pathological conditions in the RNAs’ mature form, isoforms, or precursors isolated from different liquid biopsy–obtained samples. Blood EVs miRNAs miRNA-92a and miRNA-222 are overexpressed in metastatic colorectal cancer patients associated with a lower survival rate (de Miguel Pérez et al., 2020). miR-127-3p, miR-155-5p, miR-21-5p, miR-24-3p, and let-7a-5p are increased in plasma EVs of patients with classical Hodgkin lymphoma, convenient even for detection of small residual lesions (Drees et al., 2021), while some of these miRNAs even have a potential for therapy response monitoring (van Eijndhoven et al., 2016). Urinary EVs’ isomiRs of miR-21, miR-204, and miR-375 are highly differentially expressed in prostate cancer individuals (Koppers-Lalic et al., 2016), while urinary EV-increased expressions of miR-29a-3p and miR-200a-3p are altered in patients with nephropathy in Fabry disease (Levstek et al., 2021). The altered EV miRNA levels are also reported in autoimmune diseases such as systemic lupus erythematosus, Sjogren’s syndrome, and type 1 diabetes (Michael et al., 2010; Katsiougiannis et al., 2019; Tesovnik et al., 2020; Foers et al., 2021).

Piwi-interacting RNA (piRNA) regulation molecules are produced by similar synthesis, and have similar silencing mRNA translation and protein expression mechanisms as miRNA. This species of 26–31 nucleotides long sncRNA is highly expressed in germline cells and embryos, where pi-RNAs are involved in silencing of transposable elements, the epigenetic regulation, while in adult humans, piRNAs are essential in the process of spermatogenesis. EV piRNAs were investigated in seminal plasma EVs, where piRNAs are associated with spermatogenic ability (Chen et al., 2021), while multiple myeloma-derived EV piRNAs promote tumorigenesis through re-educating endothelial cells in the tumor environment (Li et al., 2019). Vesicle-related piRNAs are also reported as biomarkers of cardiovascular disease and diabetes and even as factors with a potential involvement in cardiovascular disease development through piRNA/LINE-1/AKT, AKT, and AMPK signal pathways (Zeng et al., 2021).

Another sncRNAs reported in EVs are yRNAs, which are regulators of the DNA replication (Kowalski and Krude, 2015) and are together with RNA-binding protein Ro60 clinical important targets of autoantibodies in systemic lupus erythematosus and Sjögren’s syndrome (Boccitto and Wolin, 2019). yRNAs are in their mature form abundant in plasma immune cell-released EVs and are investigated as biomarkers. EV-altered plasma subtype yRNA ratios are reported in inflammatory disease (Driedonks et al., 2020) and carcinogenesis (Haderk et al., 2017).

EV transcriptome also includes tRNAs and tRNA fragments (tRFs), reported as highly represented in T-cell–released EVs (Gámbaro et al., 2020). Even though the cellular function of tRNA fragments is not clear, it is reported their upregulation in cellular stress and EV tRNAs can be potentially involved in target cell signaling and immune system cell regulation (Chiou et al., 2018; Tosar et al., 2020).

Similar to miRNA, other sncRNAs can be loaded into EVs together with RNA binding with Ago proteins or Y-box binding protein 1 (O’Brien et al., 2020; Ghoshal et al., 2021), which are the mediators of EV loading. The sncRNA EVs preferential directioning is reported being related to the RNA structure and nucleotide sequence motifs. Studies also report sncRNA exporting into EVs could be 5′ and 3′ nucleotide sequence specific, where miRNA sequences with specific NTA can be preferentially loaded into EVs (Koppers-Lalic et al., 2014).

In targeted cells, EV RNA molecules modulate transcription regulation *via* miRNA-like silencing mechanisms, with binding on the mRNA transcription regulatory sites, other noncoding RNAs, or intracellular receptor mechanisms. If the EV RNA is released in the cytosol of the targeted cell, the released RNA can activate RNA-sensing receptors. Besides cytosol RIG1 receptors (Boelens et al., 2014), the EV RNA can activate endosomal intracellular RNA tool–like receptors (TLR), which are also part of the antimicrobial innate immune defense system. Endosomal TLR3 can detect dsRNA motifs enriched with I:C, while TLR7 and TLR8 can detect ssRNA with enriched U and GU sequence motifs (Hornung et al., 2005, 7; Forsbach et al., 2008) in phagocytes and can transmit an activation signal to the effector cells of the immune system. How the human intracellular RNA-sensing receptors distinguish between human and foreign RNA remains unclear. The answer might be in the RNA modifications. In the literature, there are reported over 100 RNA nucleotide modifications, which can suppress or enhance the RNA stability, modify the nucleotide sequence target binding activity, or can have different affinities for the immune system regulatory receptors (Boccaletto et al., 2018; Xuan et al., 2018). tRNA nucleotide modifications have been studied to some extent. It has been reported that tRNA nucleotide sequence modifications can directly affect the tRNA structure, stability, aminoacylation, protein translation accuracy, and translation rate, and these RNA modifications associate with cell stress and cancer (Endres et al., 2019). The biological function of other sncRNA nucleotide modifications is not well understood. However *in vitro* studies show that miRNA nucleotide modifications can increase mRNA translation with the decreased miRNA regulatory function (Lockhart et al., 2018).

### EV Analysis

There are plenty of strategies for EV characterization, and they have their unique advantages and disadvantages. Therefore, a study setup with a standardization step is essential to perform a successful EV study outcome. Compared to tissues and cell cultures, the methods for detecting human liquid biopsy–isolated EVs deal with relatively small quantities of EV material. Consequently, the EV DNA and RNA material yield is often the most significant limiting factor that researchers need to challenge (Brennan et al., 2020). EV characterization and biomarker discovery include sample collection, sample processing and storage, EV isolation and characterization, EV cargo analysis, and data interpretation. All the analytical steps can induce variability in EV analysis and can have a significant impact on the final result. Therefore, it is crucial to include homogeneous groups of subjects in the study.

### EV Isolation

**FIGURE 1:**
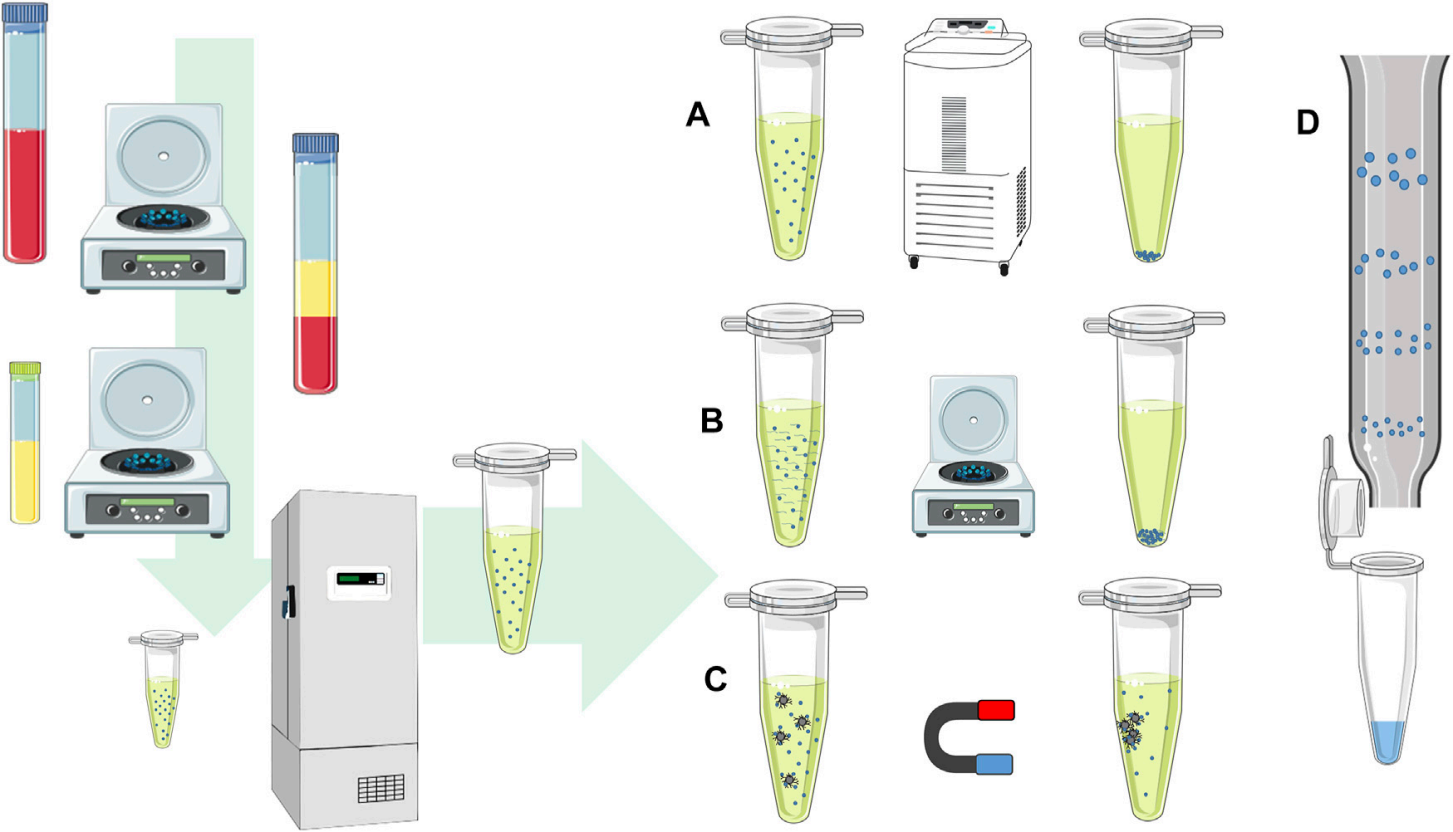
EV isolation procedure. The scheme shows the procedure for blood plasma EV isolation from the first steps after the blood collection. The samples undergo centrifugation steps to separate plasma and blood cells, while the next centrifugation step is used to remove cell debris and large protein complexes. The samples can be stored with deep freezing or further processed with methods for EV isolation. EVs can be isolated by **(A)** ultracentrifugation at high speeds, using **(B)** precipitation reagent and separated by low-speed centrifugation, **(C)** immunoprecipitation with antibodies on magnetic beads in the magnetic field, or **(D)** size exclusion chromatography based on the particle size separation on the column.

Different EV isolation strategies result in considerable differences between the EV final yield and purity (Konoshenko et al., 2018) and thus, reflect in non-reproducible EV transcriptomic and genomic profiles (Figure 1). There is no such thing as a perfect method for EV isolation. The EV isolation procedure cannot be standardized for the research applications due to the diversity of the biological samples. However, EV multiple diagnostic applications show strong tendency to standardize the EV isolation. The isolation procedure should be carefully selected in the phase of study design, and it should be adjusted to the sample type, complexity of body fluid, its volume, and required EV yield and purity to provide a sufficient EV material for the downstream analysis (Brennan et al., 2020). Due to a variety of EV procedures, the International Society for Extracellular Vesicles (ISEV) organization addressed this problem and suggested the standardized reporting of the EV isolation procedure to provide reproducible and reliable EV results (Oeyen et al., 2019)*.*

The human EV analysis starts with the human sample collection (Figure 1). In clinical practice, a least invasive sample collection is preferred. The human liquid samples of studied subjects are collected with a sterile blood draw, cerebrospinal liquid, amniotic liquid, or any other body fluids as urine or human milk (Foster et al., 2016; Bernardi and Balbi, 2020). The most commonly used starting liquid biopsy material for tracking physiological processes in a human body is the blood due to the constant environment, low-variability of human blood parameters, and relatively non-invasive collection. To preserve cells and blood plasma EV amounts in collected blood, it is advised to draw blood with anticoagulant tubes with citrate, heparin, or EDTA (Witwer et al., 2013). On the contrary, serum samples are not recommended for EV biomarkers discovery due to the platelet activation and platelet-released EVs, which can reduce the detection and sensitivity of the non–platelet derived EVs (Witwer et al., 2013; Palviainen et al., 2020). A hemolyzed and icteric sample rejection from the analysis is recommended in order to avoid sample heterogeneity, where additional protein and lipid contaminations and other cellular non-EV components can increase the amount of sample non-EV nucleotide sequences (Coumans et al., 2017). After the biological sample collection, the procedures of EV pre-isolation include multiple steps, starting with the cell removal with low speed centrifugation. In further steps, additional centrifugation at higher speeds or filtration through syringe filters is used to remove cell debris or larger protein complexes. After sample pre-processing, the processed samples are stored with deep freezing or a direct EV isolation procedure follows.

The EV isolation with an ultracentrifuge at ultra-high speeds is a gold standard for the EV isolation procedure, even though this is a time-consuming process (Momen-Heravi, 2017). Ultracentrifugation provides a low output yield, and the isolated EVs are not highly pure due to the EV co-isolation of larger protein complexes. The purity of EVs can be improved by sucrose or iodixanol gradient density ultracentrifugation, providing highly pure EV fractions with less protein contaminants, but the multiple steps of centrifugation with wash steps and EV concentration are also related to higher losses of EVs (Onódi et al., 2018; Bernardi and Balbi, 2020). Besides the impurity limitation, the obstacle of this method is often the high cost of the ultracentrifuge itself, and thus, not likely to be affordable to an average clinical research laboratory. However, there are alternatives offering more straightforward and more elegant EV isolation methods.

The precipitation reagent EV isolation is based on the addition of volume excluding polymers, where non-covalent interactions between anionic polymers and positive charge of EV membrane lipids form EV precipitates (Rider et al., 2016). The precipitated EVs are collected by centrifugation with a bench laboratory centrifuge at a lower speed up to 10.000 g. The most commonly used precipitation reagents are polyethylene glycol (PEG) polymers with different molecular masses. The PEG EV isolation yields in high EV concentrations with their wide diameter ranges in the range of exosomes, microvesicles, and small apoptotic bodies (García-Romero et al., 2019). However, with PEG, isolated EVs are co-precipitated with larger protein complexes and other non-EV particle contaminations. EVs can be isolated with commercial PEG reagent mixtures or following published protocols for PEG reagents suitable for EV isolation.

Over the past few years, there was a widespread of the EV isolation procedure with commercial size exclusion chromatography (SEC) columns, where vesicles are separated on a column by the EV diameter (Gámez-Valero et al., 2016; Lane et al., 2019). The SEC-isolated EV yields with intact EVs and with fewer contaminants than the PEG procedure and ultracentrifugation. The EV separation columns are classified for multiple uses, suitable for isolation of several samples after the wash, and column regeneration. Another similar EV isolation approach offers EV concentration or isolation with centrifugal molecular weight size cut-off spin concentrating membranes, where EV suspension concentrates on the membrane’s surface, while smaller particles and proteins are removed through the membrane (Benedikter et al., 2017; Stranska et al., 2018). Some commercial centrifugation spin membranes allow EV adsorption on the membrane surface in the presence of a binding buffer. After a wash step, the EVs are eluted or directly lysed on the membrane, and the lysate is collected for further investigation.

Tissue-specific EVs can be isolated based on specific surface proteins with immunoprecipitation, where antibodies attached on immunoprecipitation columns or magnetic beads specifically bind to EV membrane protein markers (Pedersen et al., 2017). Due to the required specific monoclonal antibodies, the isolation procedure is relatively costly and does not result in large amounts of EVs. The immunoprecipitation-isolated EVs typically do not result in high enrichment of specific EVs due to non-specific interactions with antibodies or solid-state surface interactions. There were also reports of EV isolation on liquid laminar flow microfluidic chips with the acoustic driven separation. The method has a huge potential for EV isolation with the option of EV selection by using the vesicle diameter directly from EV liquid biopsy media (Wu et al., 2017), but unfortunately, there is no commercially available isolation module on the market, yet.

All the EV isolation methods, some to a larger degree than others, also isolate non-EV impurity complexes. The most common isolated EVs from blood plasma contain EV low-density lipoproteins (LDL), high-density lipoproteins (HDL), and chylomicrones whose concentration is significantly increased in postprandial blood samples (Karimi et al., 2018). Therefore, fasting blood collection is advised for studying EVs. However, in addition to lipoprotein, even protein complexes as Ago proteins with miRNA can be co-isolated from blood plasma samples, which are not related to prandial cycles. In urine EV samples, the isolation is challenged by protein contamination with uromodulin (Tamm–Horsfall protein complex), which aggregates and forms large protein complexes (Musante et al., 2020), while synovial fluid EVs can be contaminated with glycosaminoglycan and proteoglycan complexes (Buzás et al., 2018). The extracellular non-EV RNA and DNA contamination can be degraded with RNase or DNase enzymes in pre-isolation sample treatment, while protein non-EV contaminants can be decreased by the proteinase treatment and chelating agents such as EDTA (Gutiérrez García et al., 2020; Malkin and Bratman, 2020). Before further EV processing and EV characterization nucleotide sequence extraction, it is advised to optimize and standardize the EV isolation procedure with assessment of the isolated EV average diameter and concentration, vesicle specific protein markers, and to estimate co-isolated non-EVs contaminants (Doyle and Wang, 2019). For EV reporting, the ISEV organization suggests EV detection and characterization by TEM imaging of single isolated EVs, quantification of the EV concentration, and the EV diameter using nanoparticle analysis. In addition, Western detection is appreciated to detect membrane vesicle proteins and protein contaminants (Brennan et al., 2020). The specificity of the isolated EV cargo can be characterized also by nucleotide sequence expression profiles and compare EVs with starting liquid biopsy samples, using the procedures described in the following paragraphs.

### DNA/RNA Isolation

While there are different EV isolation procedures, there are also diverse DNA or RNA isolation techniques based on different chemistry adjusted to isolate particular molecules of interest. The EV cargo typically consists of small DNA and RNA fragments; consequently, the isolation must isolate preferentially small nucleotide sequence fragments. The amount of the isolated EV DNAs or RNAs is highly variable to the isolation procedure, and it is the limiting factor that determines the EV nucleotide sequence characterization. A reasonable conclusion is that the EV DNA or RNA isolation method must ensure minimum sample losses.

In order to ensure high purity and high yield of the isolated EV RNA or DNA, it is recommended to use the standardized, commercially available reagent kits for the EV nucleotide sequence extraction. The most common RNA and DNA isolation methods include the lysis reaction with the nucleotide sequence extraction, following precipitation and purification on the silicate columns and final elution (Ali et al., 2017). In the case of RNA, the most effective isolation method is based on phenol–chloroform extraction coupled with column purification. Phenol–chloroform extraction can isolate RNA without purification columns by precipitation using carriers as linear polyacrylamide or glycogen, but precipitation isolation cannot successfully remove phenols and salts from the isolated samples. There are also less harmful, less toxic, and more environment-friendly methods, but unfortunately, these methods provide a lower yield of the isolated EV nucleotide sequences. In the last decade, EV nucleotide sequences can also be isolated by solid-phase reversible immobilization (SPRI), based on lysis, nucleotide sequence desalting precipitation, and purification on the magnetic beads (Ali et al., 2017). The method simplifies the isolation procedure, but SPRI nucleotide sequence precipitation and binding require high salt concentrations, which cannot be removed within the following wash steps successfully. Furthermore, salt impurities can interfere in the downstream analysis. A few DNA/RNA isolation procedures suggest using spike-in synthetic RNA or DNA oligos to evaluate the success of the isolation procedure and allow nucleotide sequence–related RNA quantification with the assessment of fold change trueness (Everaert et al., 2019). Because spiked-in sequences reduce the available sequencing capacity, it is not advised to use the spike-in reference in the studied samples during nucleotide sequence isolation if it is planned that the EV nucleotide sequence profile will be detected by next-generation sequencing (NGS).

**FIGURE 2:**
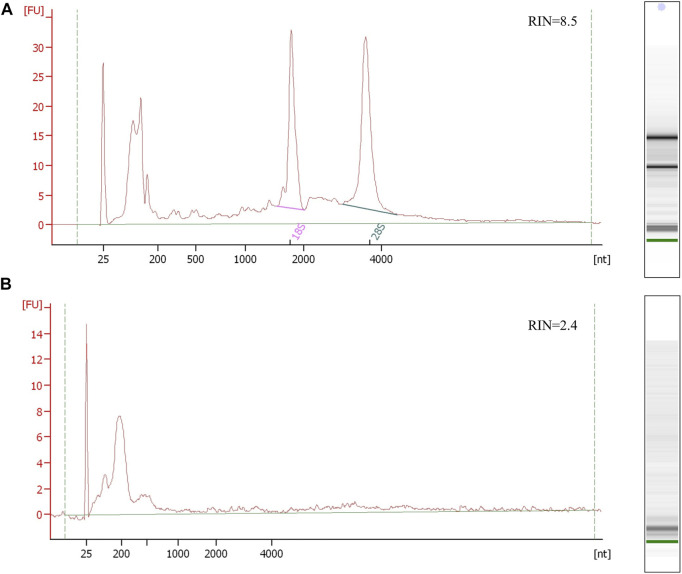
Electropherogram profiles of kidney and urine PEG-isolated EV RNA samples using the Bioanalyzer Pico 6,000 kit. **(A)** The upper electropherogram RNA profile of the kidney tissue shows the preserved RNA sample with detectable 16S and 28S rRNA and RNA integrity number (RIN) representing non-degraded RNA. **(B)** The bottom electropherogram shows urine EV sample RNA, where EVs were isolated by PEG. The RNA profile presents typical EV short RNA lengths and undetectable rRNA peaks, with unreliable sample integrity RIN due to undetectable rRNA. Both RNA samples were isolated using trizol/chloroform extraction (Qiazol, Qiagen) and commercial RNA isolation and purification columns (Qiagen RNeasy kit, Qiagen).

The EV nucleotide sequence content is still being discussed, but studies are unified in terms of a low EV nucleotide sequence quantitative amount. It was estimated that only one per 100 EVs contains miRNA (Chevillet et al., 2014; Albanese et al., 2020). However, it is hard to quantify the EV nucleotide sequence cargo due to biological sample variability, sample processing, EV isolation, and nucleotide sequence isolation losses as well as technical variability in EV nucleotide sequence detection. The amount of the isolated RNA or DNA and quality control with protein and salt contaminants can be assessed using UV-absorbance or fluorometric spectrophotometric methodology or qPCR. The isolated EV DNAs/RNAs can be additionally characterized with their size distribution and quantity, by electrophoresis on a chip. EVs’ short nucleotide sequence RNA electrophoresis profiles normally show nucleotide sequences in range up to 200 nucleotides (Figure 2). Potential 18S and 28S ribosomal rRNA peaks can be observed, but these are not specific for EVs and can indicate cell debris contamination (Tang et al., 2017). While cellular RNA and DNA quality is estimated with the RNA integrity number (RIN) and DNA integrity number (DIN), these parameters cannot evaluate the EV nucleotide sequence profile due to their short nucleotide sequence lengths.

## EV DNA/RNA Detection

Several different methods can specify EV DNA and RNA molecules. The classic approach detects EV DNA or RNA using quantitative-PCR (qPCR), where only molecules of interest are analyzed, while the technique of massively parallel sequencing allows characterization of the whole RNA or DNA EV profiles (Srinivasan et al., 2019). Due to the variability in biological samples and the technical variability of the methods of the EV nucleotide sequence detection, the EV nucleotide sequence assessment in the number of replicates of the tested condition is mandatory. While cell cultures and model organism-isolated EVs are exposed to controlled conditions and environment, the human EV molecule detection is prone to biological variability and diversity due to environment, different lifestyle and genomic heterogeneity (Srinivasan et al., 2019). Consequently, larger groups of biological replicates are endorsed to specify EV nucleotide sequence specificity related to the analyzed condition. As with all the methods in biological science, even EV detection is susceptible to technical variability; this could be estimated by technical replications and reduced by means of the use of standard commercial reagent kits and procedures.

### Quantitative PCR (Real-Time PCR)

**FIGURE 3:**
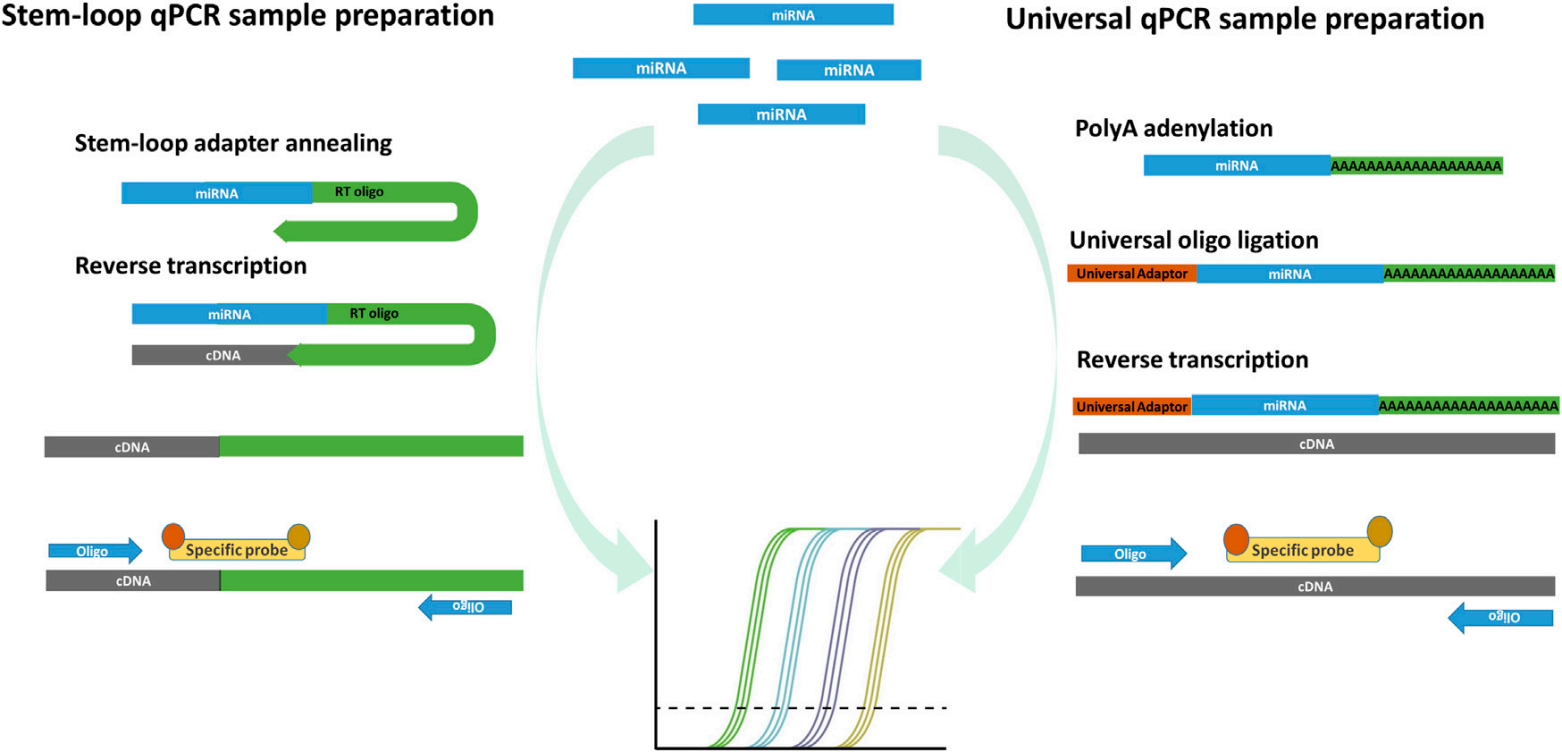
Two principles of commonly used miRNA qPCR detection. Stem-loop miRNA qPCR uses stem-loop oligos, which align on the miRNA and form the cDNA product in the step of reverse transcription. The miRNA transcripts are then amplified and detected by measuring fluorescence after the reporting probe degradation in the qPCR reaction. The stem-loop miRNA detection has a limited ability of multiplexing, requires multiple transcription reactions in which more miRNA species are detected. Universal miRNA qPCR preparation procedure transcribe all miRNA into cDNA after poly-adenylation and universal oligo ligation. The selected miRNAs are detected with specific probes capable of detecting different miRNA in multiplex reactions from the same transcription reaction.

Using quantitative PCR (qPCR), also known as real-time PCR, only the selected known sequences can be detected. EV sequences of interest can be selected based on the predetermined human organ and tissue expression data or can be predicted by known regulated pathways that are involved in the physiological and pathological conditions (Bellingham et al., 2017). The conventional RNA qPCR detection consists of the complete transcriptome reverse transcription with random hexamer oligos, following specific target amplification in DNA qPCR detection with specific oligonucleotides together with the reporting probe or reference dye in the amplification reaction. While the EV DNA and longer RNA molecules detection do not differentiate from the conventional qPCR, there are some differences in the detection of sncRNA. There are two basic qPCR approaches of miRNA detection, which are useful also for other short fragment sncRNAs detection (Figure 3). The stem-loop–mediated qPCR reaction includes a specific stem-loop reverse-transcription primer, which anneals with up to six nucleotides to the targeted miRNA and allows reverse transcription (Chen et al., 2005). The cDNA products are analyzed by PCR amplification, where specific primers align on the cDNA–miRNA complex sequence together with the reporting dye or specific probes, which complementary align on the transcribed miRNA sequence and form a detectable product and reflect the presence and quantity of miRNA. The disadvantage of the stem-loop method is the complexity of the primer design, reverse transcription variability due to stem-loop oligos, and limiting multiplexing options of different miRNA detection in the same reaction. The universal miRNA qPCR can be used to overcome the multiplexing problem and differences in the transcription efficiency. The universal miRNA and qPCR miRNA sequences are elongated by polyA polymerase, forming 3′ polyA tail, while on 5′, the universal oligo is ligated. The detection of miRNA of interest is performed by the qPCR technique, where after the reverse transcription in the PCR amplification reaction, universal oligos and specific probes are aligned on the miRNA cDNA transcript and allow its detection (Niu et al., 2015). The advantage of the universal miRNA qPCR reaction is in more uniform cDNA synthesis, which requires less sample material for multiple miRNA qPCR analyses in comparison to the loop-mediated qPCR. The qPCR EV nucleotide sequence detection allows a quick detection of the predicted differentially expressed sequences. One of the limitations of the qPCR EV nucleotide sequence detection is its data reporting, where the sequences of interest are compared to standard or reference RNAs—housekeepers, that can be selected experimentally or based on the previous results. The housekeeping RNA are independently expressed in the investigated samples regarding the variability of the studied conditions. Housekeeping RNAs allow assessing and quantifying the studied RNA with the normalized expression and relative quantification ratio. To provide convincing qPCR results, it is recommended to design an experiment with more housekeeping sequences to better verify the differential expression ratios for the studied RNAs (Gouin et al., 2017). One of the disadvantages of qPCR sncRNA detection is only mature sncRNA detection using the commercial pre-designed validated probes, while the modified sncRNA with NTA and sncRNA isoforms requires the custom probe design and PCR program standardization based on the oligo and probe melting temperature. Most qPCR methods quantify the nucleotide sequences with delta–delta–Ct and report the differential expression of the target nucleotide sequences as fold difference in the expression related to the expressed housekeeping sequence. A more precise absolute concentration of the investigated nucleotide sequence molecules can be quantitatively assessed if the qPCR measurement includes standard dilutions of known concentration by which samples’ Ct values can be translated to the nucleotide sequence concentration (Lee et al., 2020). Nevertheless, the absolute qPCR quantification approach is not used in practice. Better sensitivity and reproducibility compared to qPCR and absolute quantification can be achieved using digital qPCR (Wang et al., 2019), where sample molecules are analyzed in thousands of nanoliter-scale reactions in emulsion droplets or microchambers on a microfluidic array.

## Microarray

EV transcriptome can also be detected using a microarray, where RNA fragments are directly labeled by the fluorescent dye and hybridized on predesigned probes with antisense sequences attached on a solid-phase glass surface or chip-based media. After wash steps, the fluorescence signal detection with intensity on specific locations on a glass slide is collected with a dedicated reader. The relative fluorescence of analyzed samples is translated to data with software algorithms, which recognize and quantify the studied particular sequences on the array surface (Liu et al., 2008). With microarrays, it might be reasonable to detect EV miRNA with commercial pre-designed arrays, while mRNA sequences in human EV samples usually are not represented well enough for a successful analysis. The detection cannot distinguish between length isoforms and the detection of other sncRNAs (tRNA, siRNA, and piRNA), which requires a custom array creation. The advantage of the microarray nucleotide sequence detection is in its direct detection without PCR amplification and the uncomplicated bioinformatics analysis with microarray reader software. However, the most significant limitation of the microarray method is its requirement for a considerable amount of RNA starting material, which might not be feasible for liquid biopsy EVs.

### Next-Generation Sequencing—Second Generation

The complete EV transcriptome and EV DNA profiles can be assessed only by NGS (Amorim et al., 2017). Second-generation sequencing technology was firstly supported by Roche pyrosequencing technology and by the Applied Biosystems SOLID platform. Nowadays, the improved second generation NGS, based on sequencing by synthesis, represents the most used sequencing method, and it is offered on the market by three companies, Illumina, MGi/BGI, and IonTorrent, accessible practically to every average laboratory with different throughput sequencing machines (Hu et al., 2017). The second NGS generation allows short fragment sequencing with insert size 20–500 nucleotides at a relatively low price per read nucleotide. Many variations of the sample library preparation were developed to detect nucleotide sequences and additional sequence-related information. Both kinds of EV nucleotide sequence samples of investigation should undergo sample processing or the sample NGS library preparation procedure that includes adapter sequence ligation on 5′-phosphorylated and 3′-OH ends of sample molecules with T4 ligases. The RNA preparation procedure distinguishes from DNA library preparation in terms of the additional first step of reverse transcription, where the cDNA strand is formed. With the ligation or PCR amplification, the EV sample molecules are labeled with the index sequences (barcodes), allowing parallel multiple sample sequencing in the same sequencing run. Ligated adapter and index sequences on the sample sequences form uniform product ends, enabling uniform PCR amplification and attachment on the solid-phase surface of the flow cell in the sequencing machine. The Illumina sequencing platform as the leading sequencing technology consists of synthesis with fluorescent-labeled nucleotides, where the fluorescence of the attached nucleotides is detected by an optical system in a sequencing machine and later translated to nucleotide sequences by bioinformatics algorithms. The limitation of the second NGS generation is the limited insert size of NGS library–incorporated sequences, which is not problematic for EV sncRNA detection due to EV short sequences. An additional disadvantage presents the library processing procedure, with induced bias during the ligation step due to nucleotide sequence modifications and secondary structures in the RNA or DNA sequences (Wilusz, 2015). Additionally, the PCR step removes the information about nucleotide modifications and induce non-uniformly amplification bias of the GC-rich and homopolymeric sequences (Ross et al., 2013).

**FIGURE 4:**
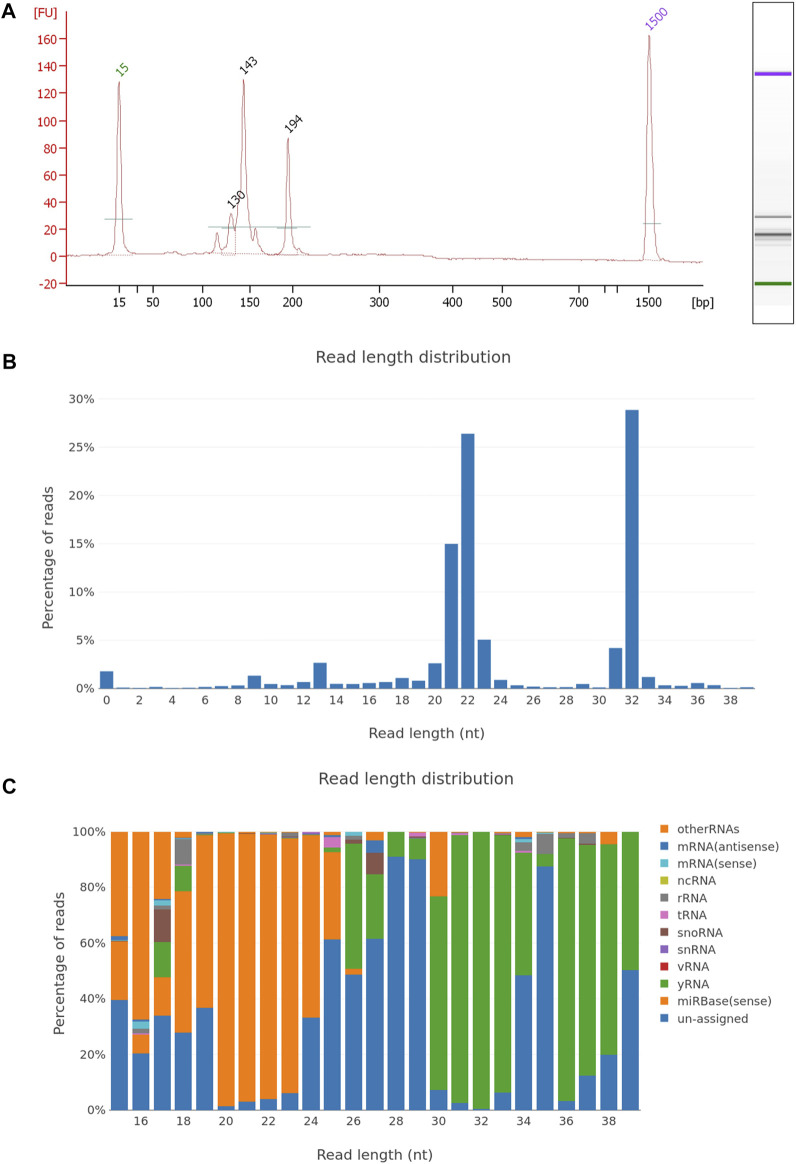
EV miRNA NGS library quality control and sequencing report. **(A)** Electropherogram of the plasma EV small RNA NGS library, with peaks of adapter dimers (120 nt), incorporated miRNA fragments (143 nt), and larger RNA inserts (194 nt). The NGS library was prepared using the NEBNext small RNA library kit and after 50cycle single-end Illumina sequencing, fastq files were generated. Sequencing data were analyzed using the sRNAtoolbox—a collection of small RNA analysis tools, where sequencing data were trimmed for NGS adapters, aligned on the human regerence genome and annotated with miRBase miRNA reference sequence data. Graphical presentation of the aligned reads is shown on graph with **(B)** read length distribution and **(C)** RNA length distribution by non-coding RNA species.

To simplify the sample sequencing preparation, several library preparation kits are on the market to prepare DNA, RNA, and sncRNA libraries. As small transcriptomic RNA molecules are abundant in EVs, it is recommended that small RNA library preparation kits are used for the NGS EV transcriptome detection (Etheridge et al., 2018; Srinivasan et al., 2019). The small RNA library preparation procedure includes the final product size selection step, where products with the incorporated miRNA or piRNA can be enriched with SPRI size selection or using gel PAGE excision of the products with specified lengths. Additionally, the size selection removes adapter dimers that are formed during the library preparation and can significantly decrease the sequencing yield (Figure 4A). Alternative small RNA library options for enriched miRNA and piRNA sequencing present tRNA/YRNA and rRNA depletion kits, which can reduce other sequences of no interest. All the NGS-prepared libraries can be enriched for particular known sequences, with the specific capture probes. Additional data improvement in the NGS detection of rare DNA or RNA molecules with a low expression can be provided by additional unique molecular identifiers (UMI), where every sequence in the sample can be marked with a specific barcoding sequence (Hong and Gresham, 2017). UMI, together with particular bioinformatics procedures, can remove PCR artefacts, decrease PCR amplification variability, and improve the detection of rear events, which are essential for an early marker detection of tumors and other diseases (Hong and Gresham, 2017; Smith et al., 2017). While different genes can be coded on the opposite DNA strands of the alleles, these alleles produce different transcripts. Strand specificity of mRNA sequencing can be achieved with RNA directional sequencing, where the degradation of non-expressed RNA PCR-formed strands produces the expression data of mRNA with overlapping genes in reverse and forward genomic sequences (Yang et al., 2017).

NGS sequencing also presents the possibility to detect epigenomic modifications, which can be tissue-specific and can be very challenging in case of EV DNA and RNA. While the DNA methylation pattern detection is supported by the commercial reagents, sample preparation requires a large amount of DNA and analysis-specialized bioinformatics pipelines (Barros-Silva et al., 2018). The DNA methylation pattern can be detected with sequencing after the bisulfite conversion of non-methylated cytosine nucleotides, which are converted to thymines (Barros-Silva et al., 2018). The alternative option of methylation nucleotide sequence detection is methylation sequence enrichment with immunoprecipitation or methylation-binding domain proteins. The detection of other nucleotide sequences’ modifications, such as oxidative stress and other reactive species–induced modifications and other amino groups, are currently still being developed and need further optimization and evaluation. The RNA modification detection is more complicated due to the lack of commercial kits and standard pipelines. While DNA commercial kits include end prep reagents for repairing 5′ and 3′ ends (Kozarewa and Turner, 2011), the commercial RNA reagent kits do not include the end repairing module. The lack of RNA repair and RNA modification causes the detection of only a fraction of the sample RNA profile (Wilusz, 2015). The non–5′-phosphoryl, remained 3′-phosphoryl groups and sequence bias can reduce the adapter ligation efficiency on RNA sample molecules. The RNA-reduced ligation efficiency can be bypassed with the application of T4 polynucleotide kinase, letter induce 3′ and 5′ end repair at sample molecules and allows further adapter molecule ligation (Sorefan et al., 2012). An additional challenge for the detection of the whole transcriptome is methylated nucleotides, which can block reverse transcription, and the RNA inserts are not included in the NGS library. The obstacle of RNA-methylated nucleotides can be neutralized by demethylation with dealkylating enzymes as α-ketoglutarate–dependent hydroxylase (AlkB) (Cozen et al., 2015). These challenges point out the importance to pay attention to nucleotide sequence modifications, which have a significant impact on the final results, even from a technical perspective.

### Next-Generation Sequencing—Third Generation

The third NGS generation is primarily designed as long-read sequencing technology for detecting DNA or RNA with several kilobase long fragments. This technology is suitable for detecting intact transcripts and providing accurate genome assemblies with successful detection even repeat sequences throughout the genome (Xiao and Zhou, 2020). Two companies offer different technologies of the third-gen NGS technology; Oxford nanopore technology (ONT) and PacBio technology. PacBio’s SMRT sequencing technology provides more accurate long-read sequencing data than ONT, but the technology requires large amounts of starting material (Xiao and Zhou, 2020); sequencing is costly, and consequently, its usage is not reasonable for short sequences mostly present in EVs. A more promising third NGS generation suitable for the EV nucleotide sequence detection is the ONT sequencing technology. A significant advantage of it is its price accessibility and its ability to detect even short nucleotide sequences with the real-time sequencing analysis together with nucleotide sequence modifications. The library preparation for ONT sequencing is similar to the second-generation sequencing, while the adapter and index molecules are attached on the 3′-end of the sample sequence together with helicase proteins, which unwinds the double-stranded native or duplicated DNA or RNA/DNA reverse transcriptase–formed hybrid complex. When unwinded nucleotide sequences are directed through protein pores on the flow-cell membrane, sequences change permeability through pores and affect the electric current that is translated to the nucleotide sequences (Xiao and Zhou, 2020; Zhang et al., 2020). The third-generation sequencing technology is under intensive development. With the improvement of sequencing hardware, sequencing chemistry, and improved library preparation, new NGS techniques will support low input material analysis applications, suitable even for EV analysis. Along with the development of bioinformatics tools, the possibility of determining EV nucleotide modifications is also expected.

## Data Analysis

Since the EV sample processing and NGS library preparation require a skilled wet lab practice, the NGS sequencing data analysis requires bioinformatics knowledge to extract sequence information and address the EV nucleotide sequence study problem. The first part of the bioinformatics analysis starts with raw data base-calling translation from sequencing signals to nucleotide sequences. The base-called sequencing files are stored in the compressed fastq files generated by the Illumina sequencing platform and fastq5 files in the case of Oxford Nanopore. The signal translation base-calling output are text files with sequencing run info, nucleotide sequences, and quality of reading nucleotides (Pereira et al., 2020).

**FIGURE 5:**
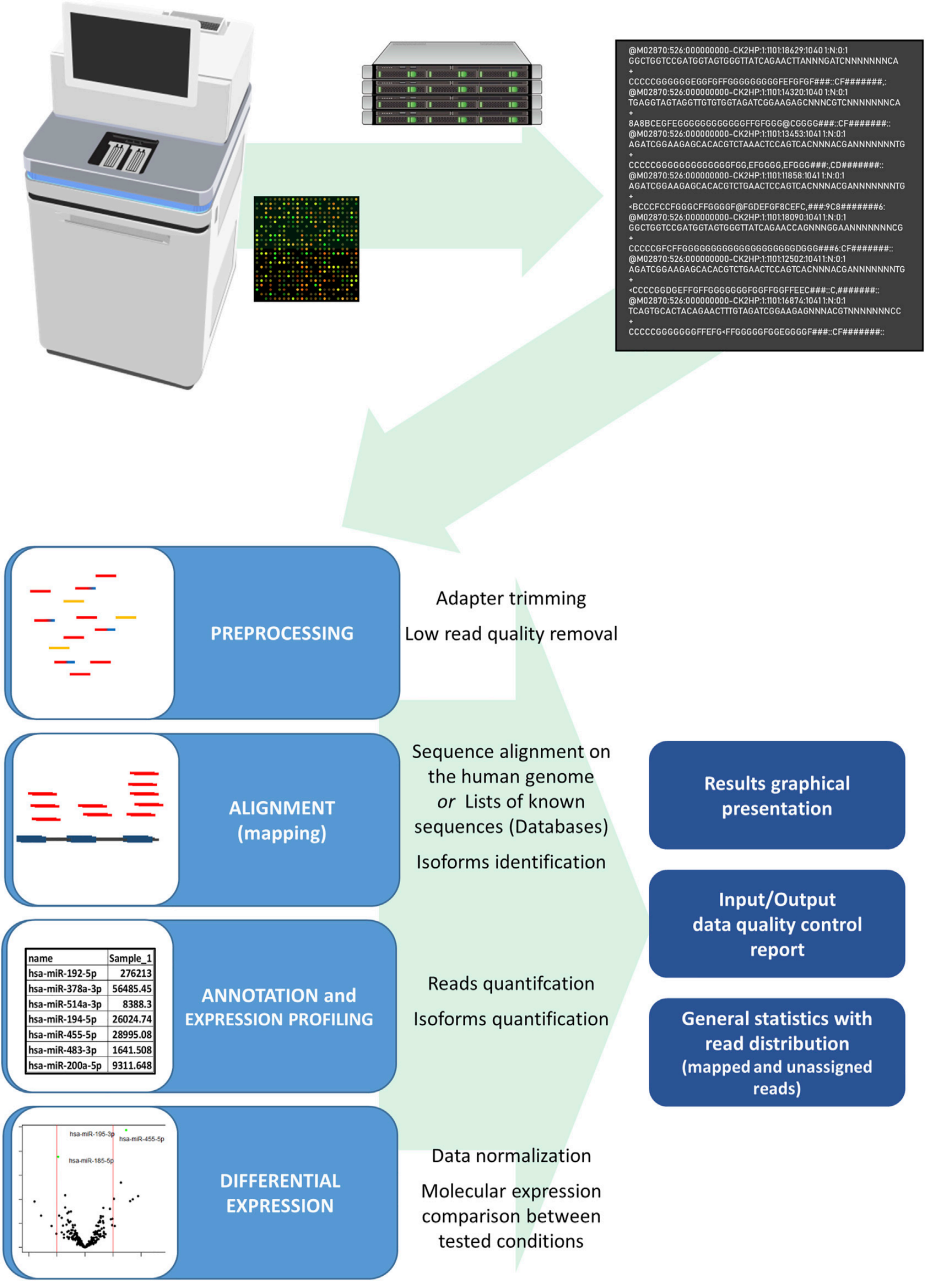
Scheme of the miRNA NGS data analysis from data generation to the final result. The Illumina sequencing platform generates bcl files, which are translated in the process of base-calling and demultiplexing by the sample index to fastq files for each studied sample. The fastq file includes sequencing run information and nucleotide sequence data with read quality data per nucleotide. In the first step of the data analysis, fastq files of the studied samples undergo the adapter and low quality sequence removal and sequence alignment on a human genome or sequences in databases follows. After alignment, annotation and quantification form a sample expression miRNA profile with the sequencing and read quality report of the studied samples. The differences in studied conditions can be identified with differential expression analysis where samples of studied conditions are compared after the expression normalization, comparison, and statistical evaluation. Final analysis results are represented in tables of normalized identified sequences, tables of differential expression with the statistical comparison, and graphical representation of read distribution and differential expression analysis results.

The RNA and DNA sequencing data analyses require different processing pipelines in the processing bioinformatics tools. However, the basic NGS pipeline includes the same basics steps: pre-processing and input data quality control, alignment, variant calling, quality control metrics report, and comparison of the expressed RNA analysis by the studied condition (Figure 5). First, low-quality sequences are removed, and other sequences with sufficient quality are trimmed for adapter sequences, which are often sequenced due to short EV sequences. After adapter trimming, sequencing reads are aligned to the reference genome (actual human genome version hg38) or known curated transcriptomic sequences, which are listed in the databases of coding and non-coding transcripts (miRBase, RNA central) (Fu and Dong, 2018). Different alignment tools are in use for the Illumina sequencing platform; for small RNA analysis, the use of a bowtie aligner is recommended, whereas for alignment of mRNA and lncRNA molecules a STAR aligner is recommended, moreover a bwa-mem aligner is used for DNA sequences. At the same time, sequences with methylation modifications require alignment with the dedicated aligner such as the Bismark aligner. After the alignment, reads are counted using Samtools and annotated with tools, where the sequences are identified with names from databases and counted by identical sequences (Fu and Dong, 2018). The result of the analyzed samples is provided as trimmed and aligned sequences with DNA or a particular RNA species, a list of genetic variants and the QC metrics report. In the study where two or more conditions are tested, the differential expression is used to detect the differences in the RNA expression in the tested groups of samples. Before the samples are compared between the studied groups, expressed sequences are normalized by statistical approaches as a ratio per total sequences or the proportion of a particular sequence compared to geometrical mean and average expression principles. To achieve consistency and reliability of differential expression results, it is advised to use more differential expression tools based on different normalization approaches as Edger, DeSeq, DeSeq2, and NOISeq (Evans et al., 2018). Developed tool packages specialized for miRNA analysis, such as sRNAtoolbox, DIANA-mAP and QuickMIRSeq, include tools for all the steps for the NGS data analysis and provide analysis results with tables of differentially expressed sequences (Figures 4B,C) (Zhao et al., 2017; Aparicio-Puerta et al., 2019; Alexiou et al., 2020). These bioinformatics tool packages are suitable also for the EV nucleotide sequence data analysis.

Like in all the steps in EV research, the NGS data analysis procedure should be reported with used bioinformatics tools and their release number. It is also required to report basic analysis parameters and raw data quality parameters, sequenced reads per sample, and raw data should be submitted to public repositories, such as the SRA server (Sequence read archive), where sequencing data are accessible publicly to other researchers. While the NGS sequencing is still costly for assessing large cohorts, it is advised to confirm the NGS findings of smaller tested groups on larger cohorts, using the qPCR method.

After the discovery and verification of the potential EV biomarkers, the biomarkers have to pass the validation on analytical and clinical levels before it can be used in clinical practice. The analytical level of novel biomarker discovery includes assessment of their accuracy, precision, analytical specificity, and sensitivity as well as analytical range, while the clinical validation determines clinical specificity, sensitivity, and positive and negative predictive values (Yekula et al., 2020). If the biomarker passes the validation stage, clinical translation follows, where the biomarkers have to pass required regulative and get the certificate of good laboratory and manufacturing practice as well as certificate for *in vitro* diagnostics (Yekula et al., 2020). First, EVs are already in diagnostic use. As first reported, the *in vitro* diagnostic EV liquid biopsy platform was presented ExoDx™ Lung (ALK) by Exosome Diagnostics. The kit detects EML4-ALK fusion transcripts in patients with non–small cell lung cancer, while the same company has also introduced exosome-based prostate cancer test ExoDx™ CE-IVD kit, which can help the physician’s decision to defer or proceed with a biopsy (McKiernan et al., 2020).

### EV Nucleotide Sequence *In Silico* Effect Analysis

The knowledge of bioactivity of specific and differentially expressed nucleotide sequences is essential to specify the effect of the investigated EV nucleotide-targeted sequences in targeted cells and organs *in vivo.* Additionally*,* the EV nucleotide sequence role is essential for conformational *in vitro* studies to promote the development of the EV-related therapeutics. Bioactive EV DNA delivered to targeted cells can be transferred to cytosol or nuclei, transcribed to RNA, and later translated in cytosol from RNA to proteins (Waldenström et al., 2012; Cai et al., 2013) and can potentially change the phenotype of the cell. Although the EV DNA and mRNA function can be predicted from the coding sequence, the non-coding RNA effect prediction requires in-depth additional analysis.

The biological EV sncRNA regulatory function can be predicted by tools that find miRNA predicted and validated targets based on the sncRNA and 3′ mRNA interaction. Some of these online or standalone tools, e.g., miRNA TargetScan (Agarwal et al., 2015), miRANDA (Enright et al., 2003), miRDB (Chen and Wang, 2020), and miRWalk (Dweep et al., 2014), predict interaction targets based on different algorithms searching for 3' or central domain mRNA–targeted complemental sncRNA sequences. Bioinformatics tools can predict the targeted antisense sncRNA-mRNA/miRNA interaction based on the organism transcriptomic data (Thorsen et al., 2012; Nguyen and Chang, 2017), and with the predicted mRNA silencing enrichment pathway analysis, the phenotypic effect can be predicted. The biological effect of differentially expressed sequences can be estimated by the pathway analysis that can be performed with freely available software, such as Cytoscape, g:Profiler, GSEA, and EnrichmentMAP, with modules for the analysis of the affected gene lists (León and Calligaris, 2017; Reimand et al., 2019). Experimentally defined differentially expressed sequences are compared to a hierarchically organized gene or RNA-expressed lists and sets of standardized terms of biological processes, molecular functions in cellular components, or tissues. The pathway analysis result is an altered cellular pathways, which can be associated with physiological and phenotypic changes (Reimand et al., 2019).

**FIGURE 6:**
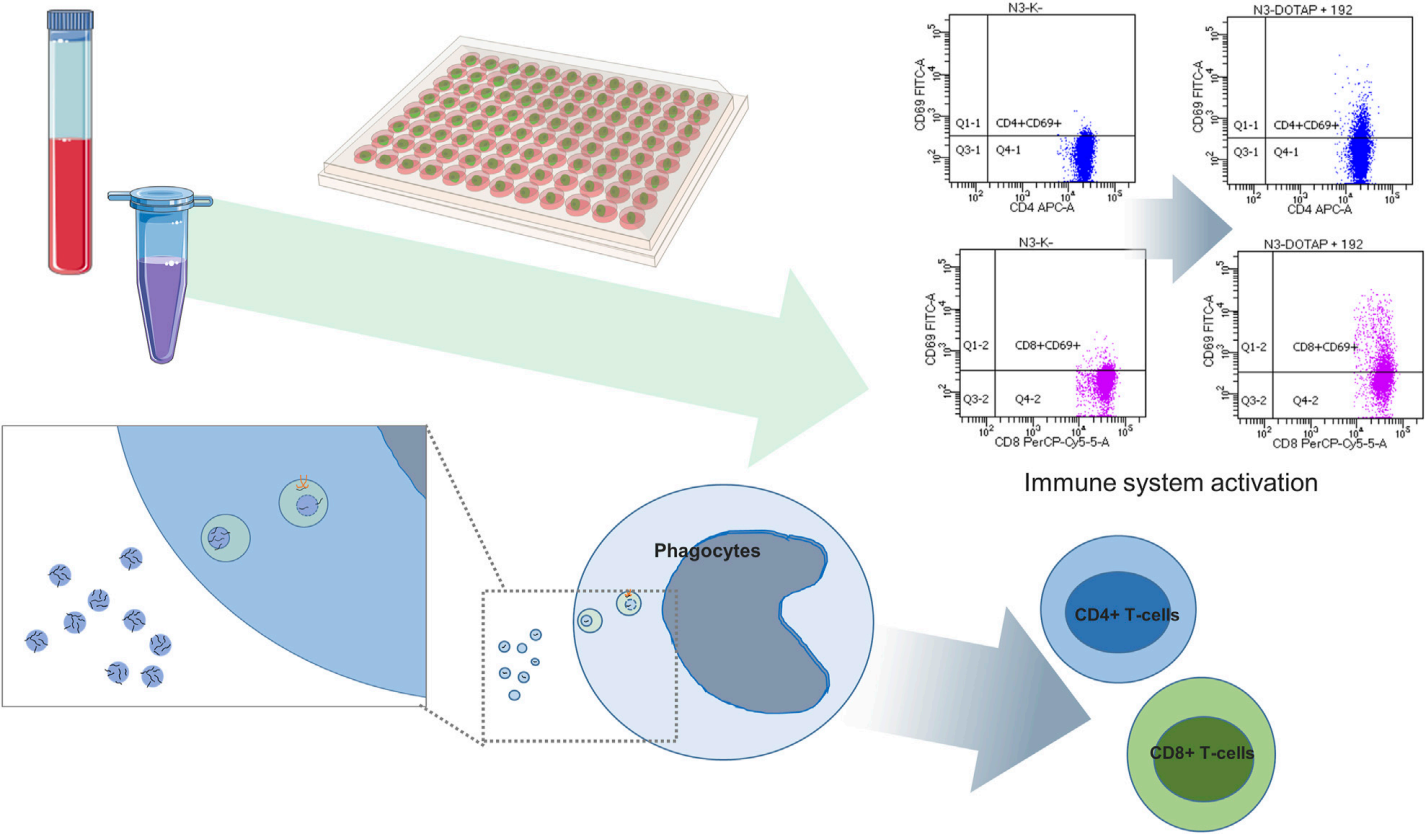
Differentially expressed EV miRNA functional study. Differentially expressed miRNAs are exposed by means of synthetic vesicles (DOTAP) to the whole blood cells of the immune system where vesicles with miRNAs are accumulated in the endolysosomal pathway of phagocytes. Vesicle-delivered miRNA overnight incubation can result in an increased expression of activation markers on effector T-cells detected using flow cytometry. The scheme is summarized work of Tesovnik et al. (2020).

The prediction tools can just estimate the EV nucleotide sequence effect on target cells, and the *in vitro* study on cell cultures or *in vivo* on animal models are essential to confirm these findings of EV nucleotide sequences in the predicted processes. Differentially expressed nucleotide sequences can be synthesized and exposed to the studied cells or organisms utilizing synthetic vesicles, and the predicted effect should be characterized by the *in vitro* or *in vivo* study, where phenotype changes can be assessed by RNA sequencing, single cell sequencing, mass spectrometry, flow cytometry, or imaging techniques. In order to estimate the immunomodulating effect of EVs delivered nucleotide sequences, the *in vitro* study can be conducted, with nucleotide sequences delivered by the endolysosomal pathway or released in the cytosol, activating nucleotide sequence–sensitive receptors (Cardarelli et al., 2016; Tesovnik et al., 2020) or changing translation and transcription (Figure 6).

### EV Nucleotide Sequences as Therapeutics

First studies of EV therapeutics show promising results as cancer therapeutics, especially in the field of cancer treatment. With the genetics and transcriptomic background knowledge of diseases, the EVs loaded with custom nucleotide sequences present enormous potential as a next-generation drug delivery platform for developing target-directed therapeutics (Herrmann et al., 2021). The use of vesicles as communication proxies’ therapeutic agents targeting specific cells is intensively investigated. Many studies investigated vesicle-delivered siRNA and miRNA for therapeutic application already in the pre-clinical trials and some even in clinical trials (Wiklander et al., 2019; Han et al., 2021). The preclinical studies show the antitumorigenic and antimetastatic activity of EVs isolated from cultured cell lines, macrophages, dendritic cells, and EV sonification loaded with small interfering RNA (siRNA) or miRNA in case of lung, ovarian cancer, and pulmonary metastasis. Nevertheless, allogenic mesenchymal stem cell EVs (exosomes) loaded with miRNA-124 are in clinical trial for treatment of cerebrovascular disorders (NCT03384433) and conditions after stroke, while KRAS G12D siRNA–loaded mesenchymal stem cell EVs are investigated for the therapeutic possibility in case of pancreatic adenocarcinoma (NCT 03608631).

Several procedures allow the production of EVs with specific nucleotide sequence loading for potential therapeutic application. The nucleotide sequence–loaded EVs can be produced *in vitro* after cell plasmid transfection, where transfected cells release EVs loaded with specific nucleotide sequences. Other EV nucleotide sequence loading options are EV incubation with nucleotide sequence suspension with physical force such as shaking, electroporation, or fractioning EVs, where nucleotide sequences can overpass the lipid membrane. EVs with specific nucleotide sequences can be also produced by gentle EV transfection with the isolated EVs and cholesterol-modified nucleotide sequence incubation or nucleotide sequence incubation with the transfection reagent. However, vesicle-like particles for nucleotide sequence delivery by the endolysosomal pathway can be also produced by transfection reagents like DOTAP and lipofectamine (Han et al., 2021). The cells or tissue *ex vivo* isolated vesicles prepared with loading procedures produce the EVs with nucleotide sequence cargo with preserved original membrane protein components and can be used only for autogenic and allogenic *in vivo* transplantation (Hocine et al., 2019; Wiklander et al., 2019). While EVs prepared from *ex vivo* cell or tissue cultures present personalized medicine application, the preparation procedures are expensive and time-consuming and cannot be used for general use due to antigen complexes. The EV non-allogenic protein content can induce immune EV rejection or can provoke the immune system destruction of cells that engulf EVs. Due to the desired general use for the entire population, therapeutic EVs with low antigen content are under development. The future of therapeutic EVs is in synthetic vesicles, which have already been used in clinical practice. Synthetic RNA lipid vesicles and technology of the lipid nanoparticle delivery system have been approved for clinical application by FDA first in 2018. ONPATTRO^®^ (patisiran) includes siRNA-targeting transthyretin, used for the treatment of polyneuropathies induced by hereditary transthyretin amyloidosis, which was first-of-its kind targeted RNA-based therapy to treat a rare disease (Hu et al., 2020). The technology became useful also for human vaccines, as the vaccine against SARS-CoV2 for preventing the SARS-CoV-19 pandemic. The synthetic vesicle platform showed the possibility for quick intervention and possible therapeutic adjustment in response to new pathogenic variants. Vesicles derived RNA with the modified nucleotides is capable of avoiding the innate immune system activation by cellular RNA-sensing receptors and promoting the expression of pathogen-like antigens against whom the adaptive immune system produces antibodies and form humoral protection against the pathogen. Vesicle delivery RNA technology is already investigated to protect against many other diseases, such as seasonal flu, respiratory viral infections (An et al., 2021), malaria, and cancer.

While the synthetic vesicles are in use for the delivery of mRNA in vaccines, the vesicles can also be loaded with human RNA to substitute cellular-damaged mRNA sequences due to genomic mutations in coding genes. Futhermore, Lainšček et al. showed EV delivery of the CRISPR/Cas system with the transcriptional regulator in the *in vivo* mice animal model, enabling activation of gene transcription (Lainšček et al., 2018). In addition, the EV therapeutic delivery by membrane protein or lipid EVs markers will allow EVs to direct the therapeutic cargo to tissues and cells, which represents the future of vesicle-delivered nucleotide sequence precision medicine.

## Discussion

**FIGURE 7:**
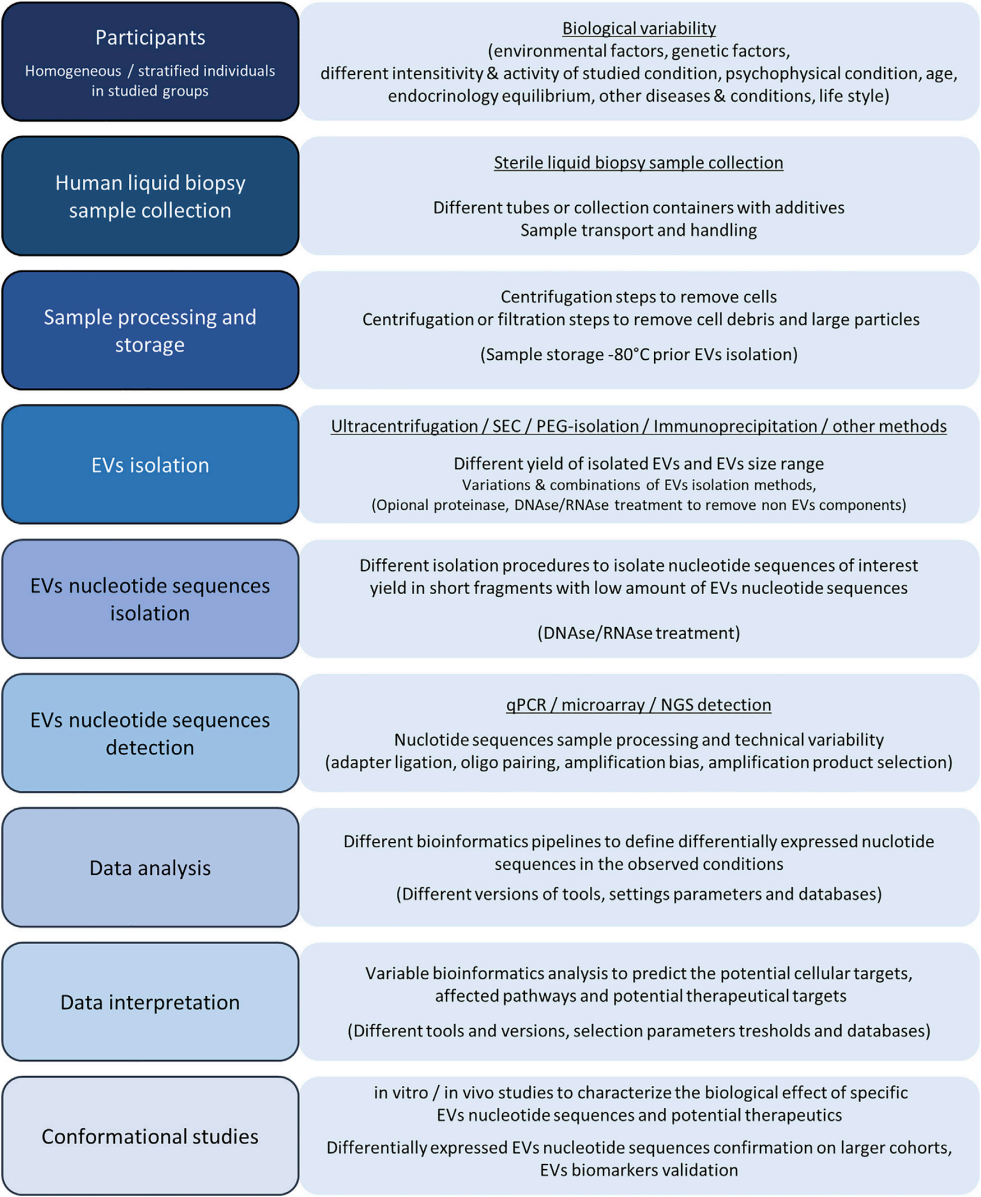
Graphical presentation of basic steps and possible variability factors in the EV processing and EV nucleotide sequence analysis. The human EV design includes the studied group participants’ selection, where biological variability factors play the main including/excluding factors for homogeneous group selection to study specific human physiological conditions. After studying the individuals’ selection, liquid biopsy samples are collected in sterile tubes or containers and processed to a cell-free liquid biopsy medium. Cells and cell debris with larger protein complexes are removed using centrifugation or filtration steps and processed samples are deep-freeze stored before further processing. EVs can be isolated using different isolation techniques and procedures, which can include protein and non-EV nucleotide sequence degradation and yield with different amounts of isolated EVs. Next, EV DNA or RNA are isolated where the nucleotide sequence profile results in a low amount of total EV DNA or RNA with length below 200 nucleotides. Isolated EV nucleotide sequences can be analyzed by qPCR, microarray, or NGS. All of the nucleotide sequence detection techniques have their advantages and disadvantages, while the limitations of all of the methods are the consequence of sequence variability and technical variation in ligation, amplification, and nucleotide pairing with complementary sequences. After the nucleotide sequence detection, the data are analyzed using bioinformatics pipelines and bioinformatics tools. A slightly more demanding analysis is required for NGS data analysis, where bioinformatics analysis parameters with the adapter trimming and sequence annotation step can have a tremendous effect on final differential expression results where data are normalized and differentially expressed EV nucleotide sequences in compared conditions are detected based on the limit parameters. Further bioinformatics data prediction analysis with databases’ information and appropriate variable thresholds can reveal the affected cellular targets, cellular pathways, and EV nucleotide sequences as therapeutic targets, which can be further characterized and confirmed within *in vitro* or/and *in vivo* studies. Differentially expressed EVs are also potential biomarkers, while their diagnostic potential must be confirmed with additional experiments on larger cohorts and validation steps.

From the first EV reports, where EVs were assumed as cell-released garbage, the EVs research revealed the complexity of EVs role in intracellular signaling and cell communication with their potential as biomarkers of physiological states and pathogenesis. In humans, most studies are focused on intracellular communication, even though EVs are also involved in the interkingdom vesicle communication between human cells and microorganisms or parasites. Gut bacterial microbiota EVs in the human organism can modulate metabolism, endocrine response, and consequently regulate the entire human organism with pro-inflammatory or anti-inflammatory response (Ahmadi Badi et al., 2017; Macia et al., 2019). Similarly, viral infection–released EVs with viral components can increase human cell susceptibility to infections and promote cell infection rate, but on the other hand, they can deliver viral molecules to cells of the immune system and act as messengers promoting the immune system response and viral infection neutralization (Urbanelli et al., 2019; Martins and Alves, 2020). In the last two decades, the technical and methodological breakthrough in genomics and transcriptomics resulted in accessible massive nucleotide sequence detection. Consequently, the breakthrough in genomics and transcriptomics became one of the most accessible and prospective research fields, even for studying EVs. Plenty of EV methods and approaches are available and published for the EV characterization. Still, unique human liquid biopsy samples together with a low amount of the isolated EVs are the most challenging and limiting factors in human EV research. An additional problem in EV research is EV methodological variability, causing incomparability of the published results and promoting a need for protocol standardization and validation of the results characterizing the EV-related nucleotide sequences (Figure 7). Accordingly, special care and a critical view are required to collect findings from multiple studies with a similar research topic and to draw conclusions about specific EV features.

More and more EV studies implement NGS in their study strategy and consequently, precise sample metadata annotation is imperative for reliable interpretation of EV-expressed nucleotide sequences. While most studies report *in silico* differentially expressed EV-nucleotide sequences’ role in the organism, the excat biological function of these differentially expressed nucleotide sequences is often not verified with *in vitro* or *in vivo* experiments. In the future, more studies will try to characterize the EV nucleotide sequence biological effect. By collecting data from standardized compatible experimental setups, it would be possible to build deep learning algorithms and artificial intelligence in order to predict pathological differences. EVs automated processing and EV deep sequencing will make possible detection of pathological abnormalities even at early stages of the disease before the first clinical symptoms appear. With these conditions, the EVs would become reliable diagnostic markers and supportive diagnostic tools in clinical practice, driving the development of novel therapeutic approaches. The exciting field of EV nucleotide sequences research has a bright future. Advanced NGS methods with single molecule resolution will give RNA and DNA nucleotide sequences a whole new perspective and additional dimension by identification of nucleotide sequence modifications. Nowadays, the EV nucleotide sequences are commonly investigated only by their primary nucleotide sequence. While DNA nucleotide modifications can lead to gene silencing and mutation development, RNA modifications can have an essential role in cellular function regulation and immunomodulation and could be associated with wide plethora of diseases. Just as the presence of EV DNA modifications is not well understood, the same goes with the EV RNA modifications, although intracellular mechanisms of pathogen detection imply their potential function. The knowledge of the role of EV nucleotide sequence modifications will gain importance in the upcoming years and will probably reveal an additional regulatory layer of multiple biological functions and disease pathologies.

The technology and methods in genetics are well under development, and the innovations in the methodological procedures will support low EV nucleotide sequences material input. Since the cellular nucleotide characterization techniques aim to define the expression profiles on a single cell level, similar characterization would be reasonably appreciated in EVs. Single vesicle nucleotide sequences with a simultaneous surface protein markers approach would give a whole new perspective on the EV’s complexity and diversity. Compared to present studies, single vesicle information would directly link the EV nucleotide sequences and proteomic profile to the EV-released tissues. Such an approach will allow the generation of EV multidimensional multi-omics data and facilitate a new specific biomarker discovery. Determining the roles of molecules inside vesicles and those on their surface represents another challenge for future characterization. The EV research is focused on the characterization of EV lumen nucleotide sequences, even though EVs can contain parental cell origin RNA and DNA attached on the EVs’ surface lipids or anchored by glycosylated anchors (Flynn et al., 2021).

Because of the regulatory role of EVs, their therapeutic possibilities are being discussed and some preclinical and clinical trials already show promising results. Autologous platelet-produced therapeutic EVs significantly improve chronic postoperative healing and regenerative medicine (Vozel et al., 2021), while mesenchymal stem cell-derived EVs are investigated in diverse intervention use (Klyachko et al., 2020). Therapeutic platelet or mesenchymal stem cell-derived EVs with their cell growth factors and nucleotide sequence cargo promote cell proliferation, fibroblast migration, angiogenesis, and anti-inflammatory response in targeted cells, thus promoting tissue, bone, and muscle repair and neuroregenerative response (Antich-Rosselló et al., 2021; Lee and Kim, 2021). Despite all the regenerative potential, EVs are not commonly used in clinical practice due to immunological limitations and other biological restrictions of their use. Since the human and cell-derived and modified EVs procurement are time and money consuming, the artificial lipid nanoparticles with vesicle properties without unwanted immunogenic components are becoming the therapeutics of the modern age. The COVID mRNA vaccines, as the first modern vesicle-RNA vaccines, showed a great potential of vesicles as delivery media, not only for mRNA, but also for other therapeutics. Therapeutic vesicles may contain different therapeutic cargo, from metabolites, cytostatic agents, DNA and RNA nucleotide sequences, or the CRISPR/Cas system for transcriptional regulation. Almost unlimited possibility of synthetic EV-loading technology has an opportunity to treat injuries, in manner to improve regeneration, and treat diseases from cancer, autoimmune disorders, such as lupus, rheumatic diseases, multiple sclerosis etc. Additionally, therapeutic EVs have a potential in genetic disorder treatment, from alleviating the disease symptoms or even eliminating the cause of the diseases. As studies show the EV option of loading with Cas9, whose cleavage specificity is guided by sgRNA, EVs could also become targeted cell genomic editing tools. Such Cas9-loaded vesicles might be used for *ex vivo* or even *in vivo* treatment for inborn genetic disorders and could substitute *ex vivo* cell treatment for somatic mutations with viral vectors. While the lipid nanoparticles are preferentially accumulated in the lymph nodes, spleen and lungs, the future of EV therapeutics is in specific EV target tissue delivery. This could thus be achieved by EV-specific target membrane surface proteins (Charoenviriyakul et al., 2018), lipids, and glycans content. The universal synthetic EVs, without immunogenic components and target tissue delivery with nucleotide sequences, present a very promising therapeutic potential for the future.

EVs and their nucleotide sequences, with their diversity, present an exciting research field that is still practically undiscovered and with enormous innovation potential represent one of the most promising fields of biomedical research.
